# Supplementary material for: Association between Serum Levels of Interleukin-25/Thymic Stromal Lymphopoietin and the Risk of Exacerbation of Chronic Obstructive Pulmonary Disease
Source: Biomolecules. 2023 Mar 20;13(3):564. doi: 10.3390/biom13030564 (PMC10045988; doi:10.3390/biom13030564)
Supplement: Supplementary file 1 [file biomolecules-13-00564-s001.zip › biomolecules-2233384-supplementary.pdf]

Supplementary Material

Table S1. Differences in clinical characteristics according to the breakdown of IL-25 and TSLP values

	Both low (n=113)	IL-25 low & TSLP high (n=99)	IL-25 high & TSLP low (n=150)	Both high (n=200)	P-value
Age	67.5±7.6	68.1±7.1	69.5±7.9	69.3±7.6	0.109
Sex (male)	110 (97.3%)	93 (93.9%)	146 (97.3%)	195 (97.5%)	0.365
Smoking history					0.081
- Former smoker	69 (61.1%)	69 (69.7%)	112 (74.7%)	146 (73.0%)	
- Current smoker	44 (38.9%)	30 (30.3%)	38 (25.3%)	54 (27.0%)	
BMI (kg/m^2)	23.6±3.4	23.3±3.0	22.9±3.2	23.0±3.2	0.287
mMRC score					
SGRQ score	33.4±20.1	29.0±17.9	32.6±19.3	27.6±17.3	0.017
CAT score	14.1±8.2	14.3±7.7	14.7±7.8	13.2±7.9	0.326
6MWT (m)	417.7±110.7	428.1±100.9	387.6±93.7	410.8±114.9	0.039
Hx of asthma	22 (20.0%)	27 (28.1%)	34 (22.8%)	48 (24.1%)	0.581

Physician diagnosed ACO	27 (27.0%)	16 (19.3%)	21 (18.1%)	33 (20.2%)	0.398
FEV1 (L)	1.9±0.6	2.0±0.6	1.8±0.6	1.9±0.6	0.399
FEV1 (%)	65.6±18.7	65.5±17.6	63.2±20.3	65.6±18.1	0.626
FVC (L)	3.5±0.7	3.6±0.8	3.5±0.8	3.6±0.7	0.825
FVC (%)	84.7±14.2	86.4±16.3	85.2±16.4	85.6±14.1	0.859
FEV1/FVC	53.3±11.4	54.0±11.4	50.2±12.0	52.7±11.2	0.043
DLco (mL/mmHg/min)	67.0±17.3	68.5±19.0	63.5±19.3	62.8±17.8	0.052
Blood eosinophil count (/mm^3)	209.6±166.4	266.7±367.8	203.7±182.3	234.7±262.7	0.239
IgE (mg/dl)	232.9±285.6	187.9±229.4	213.0±238.9	222.9±238.0	0.692
FeNO (ppb)	25.4±17.6	29.3±16.4	26.9±15.2	27.0±18.7	0.902
Hx of exacerbation	13 (11.7%)	14 (14.3%)	15 (10.2%)	29 (14.9%)	0.586
IL-25 (ng/ml)	1.2±0.1	1.5±0.1	1.2±0.1	1.5±0.6	<0.01

TSLP (pg/ml)	0.4±0.2	0.5±0.2	1.0±0.3	1.1±1.0	<0.01

Table S2. Risk of moderate-to-severe exacerbations between IL-25 high/TSLP high and both low group.

	Moderate-to-severe exacerbation		
	OR	95%CI	p-value
IL-25 high & TSLP high (vs both low)	0.90	0.77-1.06	0.21
